# Advances in the Role and Mechanisms of Essential Oils and Plant Extracts as Natural Preservatives to Extend the Postharvest Shelf Life of Edible Mushrooms

**DOI:** 10.3390/foods12040801

**Published:** 2023-02-13

**Authors:** Yuxi Guo, Xuefeng Chen, Pin Gong, Ruotong Wang, Aoyang Han, Zhenfang Deng, Zhuoya Qi, Hui Long, Jiating Wang, Wenbo Yao, Wenjuan Yang, Jing Wang, Nan Li

**Affiliations:** School of Food and Biological Engineering, Shaanxi University of Science and Technology, Xi’an 710021, China

**Keywords:** edible mushrooms, essential oils, plant extracts, postharvest, mechanism

## Abstract

China has a large variety of edible mushrooms and ranks first in the world in terms of production and variety. Nevertheless, due to their high moisture content and rapid respiration rate, they experience constant quality deterioration, browning of color, loss of moisture, changes in texture, increases in microbial populations, and loss of nutrition and flavor during postharvest storage. Therefore, this paper reviews the effects of essential oils and plant extracts on the preservation of edible mushrooms and summarizes their mechanisms of action to better understand their effects during the storage of mushrooms. The quality degradation process of edible mushrooms is complex and influenced by internal and external factors. Essential oils and plant extracts are considered environmentally friendly preservation methods for better postharvest quality. This review aims to provide a reference for the development of new green and safe preservation and provides research directions for the postharvest processing and product development of edible mushrooms.

## 1. Introduction

Edible mushrooms are a delicious and healthy food owing to their richness in protein, amino acids, and polysaccharides [1,2,3]. The global production of edible mushrooms is now largely stable, and with the development of the edible mushroom industry, their world trade is also increasing, contributing widely to human living standards [4]. China is one of the world’s largest producers and exporters of wild edible mushrooms [5]. The value of cultivated mushroom production ranks in the top five after grain, vegetable, fruit, and edible oil plantations and is higher than the sugar, cotton, and tobacco industries [6]. However, due to the high water content of edible mushrooms, the respiratory and metabolic rates are fast and the tissue of the seeds is tender and susceptible to mechanical damage and microbial contamination during the storage process, resulting in browning and quality decline [7,8,9,10].

Various preservation strategies are widely used to extend the shelf life of mushrooms. Among these are various physical treatments, including irradiation [11], atmosphere packaging [12,13,14], ultrasonication [11,15], high-voltage electric field treatment [16,17], and pulsed light treatment [18,19,20]. It is well known that operational complexity and energy consumption issues are important factors limiting the industrial application of these traditional physical methods. For instance, radiation treatment places high demands on the operator’s technical requirements and safety assessment, while gas conditioning treatment involves high concentrations of CO_2_, with potential CO_2_ hazard risks for the operator. Traditional chemical methods (use of preservatives for freshness) [21,22] are gradually creating challenges in terms of consumer acceptance because of their potential negative impact on the environment and human health [23]. To develop safe, environmentally friendly, and effective techniques for maintaining the postharvest quality of edible mushrooms, a great deal of research has been carried out in the last five years, and many review articles have been published [9,10,24,25,26,27]. Natural substances with recognized antibacterial and antioxidant properties have been considered for use in the formulation of edible coatings to extend shelf life [28,29]. Essential oils (EOs) and plant extracts are generally recognized as safe (GRAS) and have a low resistance-inducing effect on a range of microorganisms [23,30,31,32]. EOs are aromatic oil-like liquids derived from plants. Plant extracts can act in a pleiotropic manner on several cellular and molecular targets [23]. They are an excellent postharvest preservative for edible mushrooms due to their high antibacterial properties and refreshing aroma; however, a summary of the specific mechanisms of action of plant extracts and EOs on edible mushrooms is lacking.

This paper reviews and summarizes the mechanisms of action of EOs and plant extracts on the preservation of edible mushrooms and their applications to provide a theoretical basis for new strategies in preserving edible mushrooms and a reference for the development of green storage processes.

## 2. Factors Influencing Postharvest Spoilage of Edible Mushrooms

The quality degradation of edible mushrooms occurs after harvesting, which can seriously affect their commercial value and shelf life. Among the most significant quality changes are moisture loss, browning, alteration of hardness (softening), and loss of nutritional and flavor qualities. At the postharvest stage, the quality of edible mushrooms is highly correlated with internal factors (including water activity, respiration rate, and microbial activity) and external factors (including storage temperature, relative humidity, and mechanical damage). This paper summarizes the internal and external factors affecting the postharvest storage quality of edible mushrooms.

### 2.1. Internal Factors

#### 2.1.1. Moisture Activity

**Figure 1:**
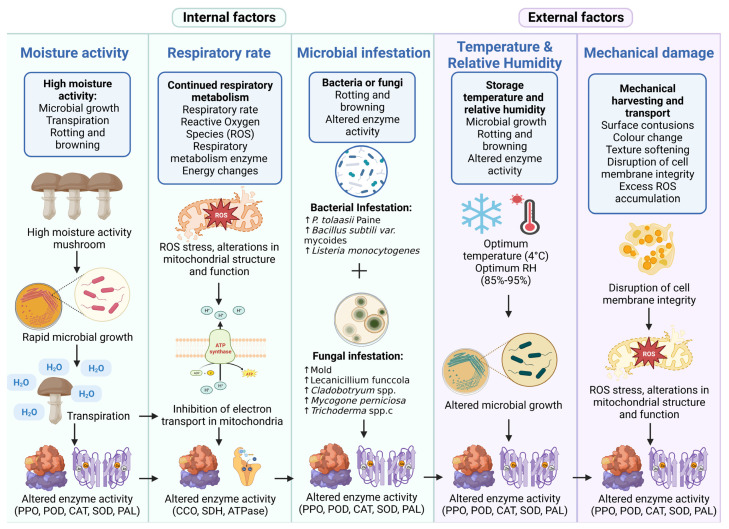
Factors influencing postharvest spoilage of edible mushrooms. (PPO: polyphenol oxidase; POD: peroxidase; CAT: catalase; SOD: superoxide dismutase; PAL: L-phenylalanine ammonia-lyase; CCO: cytochrome C oxidase; SDH: succinate dehydrogenase; ROS: reactive oxygen species.) Created with BioRender.com.

Moisture activity is the ratio of the equilibrium water vapor pressure (P_w_, kPa) of a foodstuff to the saturated vapor pressure of pure water (P_wo_, kPa) at the same temperature. It reflects the degree to which moisture is bound to the food (the degree of freeing) and is affected by lipid oxidation, microbial growth, enzymatic and non-enzymatic activity, and changes in the texture profile [33]. Water activity is, therefore, an important factor in measuring the storage quality of edible mushrooms [34]. In contrast to other crops, freshly harvested mushrooms have high moisture content [35,36], leading to the rapid growth of microorganisms [37]. Meanwhile, edible mushrooms are susceptible to quality deterioration such as moisture loss, wilting, decay, and browning during storage due to their unprotected epidermal structure, transpiration, and high polyphenol oxidase (PPO) activity [38] (Figure 1).

#### 2.1.2. Respiration Rate

In respiration, O_2_ levels influence respiratory metabolic processes, and the postharvest storage quality of edible mushrooms depends on the respiration rate [10,39]. The maintenance of a relatively low respiration rate, therefore, plays a vital role in extending the shelf life of edible mushrooms [40,41,42]. The respiration rate of mushrooms is influenced by the storage temperature and time [43]. In general, the higher the storage temperature, the higher the respiration rate of postharvest mushrooms [44]. Edible mushrooms have a high respiration rate (132–158 and 20–35 mL CO_2_ kg·h^−1^ at 20 °C and 5 °C, respectively) due to their thin and porous epidermal structure [44]. Microbial spoilage increases during postharvest mushroom storage, promoting increased respiration rates, dehydration, browning, and microbial growth due to physiological stress from microbial or pest infestations [45]. In addition, the postharvest storage process is an abiotic stress on mushrooms, leading to the inhibition of electron transfer in the mitochondria and an increase in the production of reactive oxygen species (ROS) [45]. When ROS levels exceed the cell’s antioxidant capacity, oxidative stress occurs and causes damage to lipids, membranes, proteins, and DNA, which is mediated by cellular structures (e.g., mitochondria) [40,46,47,48]. Typically, respiration directly affects changes in mitochondrial membrane enzymes associated with respiratory metabolism, such as cytochrome C oxidase (CCO) and succinate dehydrogenase (SDH) (which are usually inhibited as respiration increases) [49,50,51,52] (Figure 1).

#### 2.1.3. Microbial Infestations

During the postharvest period, edible mushrooms are exposed to a wide range of microorganisms, such as bacteria and fungi, due to their lack of protective epidermal tissue and high water content, resulting in many changes in enzyme activity [53] (Figure 1). Bacteria such as *Pseudomonas tolaasii* [54,55,56], *Bacillus subtili* [57,58], *Pseudomonas fluoresens* [13,54,59,60,61], and *Listeria monocytogenes* [57,58,61,62] are the main bacteria infecting edible mushrooms. Additionally, mold is another microorganism that can cause infection in edible mushrooms [10,57,63,64]. The negative effects of diseases caused by fungi such as *Lecanicillium funccola* [65], *Cladobotryum* spp., *Mycogone perniciosa*, and *Trichoderma* spp. also limit yields and harvest quality [66]. During physiological stress from microbial or pest infestations, peroxidase (POD) and catalase (CAT) are activated [67,68,69], leading to adverse changes in plant texture, taste, or odor and possibly to browning [44,70,71] (Figure 1).

### 2.2. External Factors

#### 2.2.1. Temperature and Relative Humidity

Temperature and relative humidity play an important role in the growth of microorganisms and are also the main factors influencing the browning of mushrooms [72,73]. The various nutrients (polysaccharides, aldehydes, and phenolic compounds), quality characteristics, and microbial reproduction in mushrooms are influenced by temperature [52,73]. Generally, increasing the storage temperature accelerates the aging, browning, weight loss, and texture softening of mushrooms. Low temperatures are recommended for the postharvest storage of mushrooms. The optimum storage temperature for edible mushrooms is generally around 4 °C, but cold damage can occur if the storage temperature is below 0 °C [73,74]. In addition, the temperature can affect the enzyme activity and, thus, the spoilage of mushrooms [73]. For example, the amount of ascorbic acid depends on the intensity of the oxidation process of ascorbate oxidase caused by enzymatic activity, and the spoilage and ripening of the crop is strongly influenced by it, while the ascorbic acid concentration in the plant depends on the storage time, the temperature during storage, and the amount of oxygen in the atmosphere [75] (Figure 1). Relative humidity (RH) significantly affects moisture loss in mushrooms; the lower the RH, the faster the moisture loss. The use of a higher RH under storage conditions helps to minimize postharvest mushroom quality losses [9,14]. A high RH (85%–95%) should be maintained in all cases [9,76] (Figure 1).

#### 2.2.2. Mechanical Damage

Mushrooms do not have a cuticle to protect them from physical or microbial attack and moisture loss [9]. Therefore, they are susceptible to mechanical damage and a reduced shelf life during postharvest handling and various modes of transportation [37]. Mechanical damage caused by postharvest mushroom cutting treatments can disrupt the integrity of the cell membrane and induce the production of excess ROS. Elevated levels of ROS may lead to membrane lipid peroxidation, resulting in a loss of membrane integrity and cellular compartmentalization. Moreover, this allows the PPO in the plastids to mix with the phenols present in the vesicles, which manifests as browning [77] (Figure 1).

## 3. Mechanism of Action and Application of EOs and Plant Extracts for the Preservation of Edible Mushrooms

Edible films and coatings are biopolymer-based packaging materials that may be consumed after food application. They are manufactured by homogenizing a composite aqueous solution in the presence of a plasticizer, followed by casting and evaporation of the water. Plant extracts can also be easily incorporated to produce a biologically active form of “active packaging”. Moreover, plant extracts are often used in edible films because they are generally safe at low concentrations.

In recent years, with the increasing consumption of edible mushrooms worldwide, the demand for improvements in their quality has led to the widespread use of environmentally and economically friendly methods in the preservation of edible mushrooms. In this regard, the impact of EOs and plant extracts in extending the shelf life of edible mushrooms holds great promise.

### 3.1. Application of EOs in Edible Mushroom Preservation

**Figure 2:**
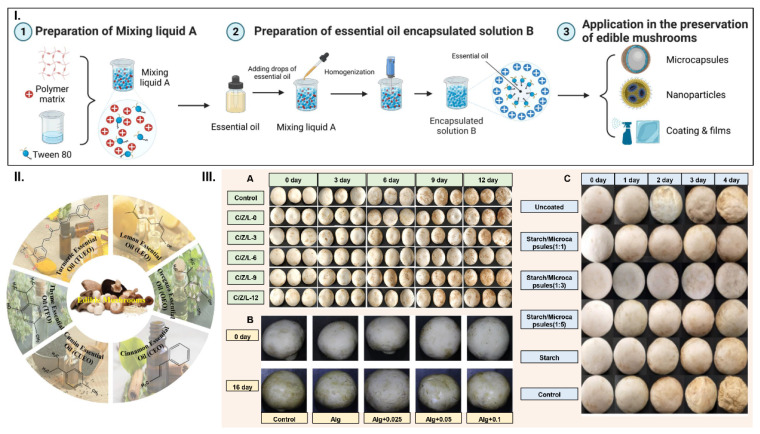
Forms of essential oils applied to the preservation of edible mushrooms ((**I**) Schematic diagram of the formation of an essential oil slow-release system and its application form in the preservation of edible mushrooms; (**II**) the main components of essential oils used to preserve edible mushrooms; (**III**) changes in edible mushroom quality after essential oil treatment. (**A**) *Agaricus bisporus* after chitosan/zeaxolysin/lemon essential oil composite film treatment [70]; (**B**) *Agaricus bisporus* after treatment with cinnamon essential oil [84]; (**C**) *Agaricus bisporus* after starch/cinnamon essential oil composite film treatment [85]. Copyright 2021, Elsevier.) Figure 2A created with BioRender.com.

Plant EOs are commonly found in all parts of plants, including the roots, trunks, bark, stems, leaves, flowers, and fruits. They can be considered as potential alternatives to synthetic agents such as butyl-hydroxytoluene, which is classified as GRAS and approved by the US Food and Drug Administration [78]. EOs may contain terpenes, aldehydes, fatty acids, phenols, ketones, esters, and alcohols with food-preserving effects [79]. They have broad-spectrum antibacterial activity and excellent antioxidant properties; some also have a refreshing aroma [80,81]. Due to their volatility, EOs only last a short time [82], but they can be encapsulated in a slow-release system for their sustained release [79,83] (Figure 2, Table 1).

#### 3.1.1. Lemon Essential Oil (LEO)

LEO is extracted from citrus lemons and is used as a food preservative, flavoring agent, and preservative due to its good antioxidant and antibacterial properties [103]. Wang et al. incorporated various concentrations of LEO into chitosan/zeaxolysin composite membranes (C/Z/L membranes), which had strong antibacterial and antioxidant activities due to inhibiting the respiration rate and microbial growth, to slow down the aging process of *Agaricus bisporus* and maintain postharvest quality [70]. It was seen that the addition of LEO effectively increased the antioxidant and antimicrobial activities of C/Z films. Throughout the storage period (4 °C, 12 d), mushrooms packed in films with 6% LEO exhibited the lowest browning index and respiration rate, effectively maintained antioxidant capacity, improved tissue hardness, and inhibited the browning process by inhibiting PPO and POD activities. At the same time, inhibition experiments showed that C/Z/L films exhibited good inhibition activity against food-borne pathogenic bacteria (*Escherichia coli* and *Staphylococcus aureus*) [70].

#### 3.1.2. Oregano Essential Oil (OEO)

The main component of OEO is carvacrol, which has excellent biological activities, such as antibacterial and antioxidant properties [104,105,106]. Due to its importance in the food, pharmaceutical, and cosmetic industries, the demand for OEO has been growing steadily in the global market [107]. Salgueiro et al. reported that OEO completely inhibited the growth of *Candida albicans* at a concentration of 0.25 mg/mL and was also fungicidal against *S. aureus.* In addition, the phenolic hydroxyl group of carvacrol could act as a donor for peroxide radicals during oxidation, preventing lipid peroxidation chain reactions and protecting lipids from oxidation [108,109]. Cui et al. prepared OEO-MSNPs/SA films at different concentrations by embedding OEO into mesoporous silica nanoparticles (MSNPs) and incorporating them into sodium alginate (SA), which exhibited strong intermolecular interactions through hydrogen bonding, resulting in biofilms with excellent mechanical and water resistance properties, as well as antioxidant and antibacterial activities [105]. A recent study by Lu et al. showed that SA membranes with 1.0 wt% OEO-MSNPs had the highest antioxidant capacity in 95% ethanol food simulants, with an oxidation inhibition rate of 75.31%. SA membranes with 1.0 wt% OEO-MSNPs showed significant antibacterial activity against *Curvularia lunata*. It was seen that the higher the content of OEO-MSNPs, the greater the inhibition of microbial penetration and the better the storage and preservation of edible mushrooms. However, the reduced transparency means a tradeoff with the enhanced UV-blocking properties, and the decreased elasticity may lead to a restricted application scenario [90]. Further testing in this area should still be carried out to assess the suitability of the market packaging.

#### 3.1.3. Cinnamon Essential Oil (CEO)

CEO is a natural volatile oil with strong antibacterial activity and is more widely used to preserve edible mushrooms [85,101,110,111,112]. Gao et al. demonstrated that the fumigation of mushrooms with appropriate doses of CEO could reduce the rate of cap opening and inhibit the aging of mushrooms [111]. In terms of protein regulation, cinnamaldehyde (a component of CEO) can inhibit the transcription of effector and regulatory proteins [113]. Cinnamaldehyde has excellent antibacterial properties, altering the distribution of lipids in the cell membranes of pathogenic bacteria and disrupting them [114]. It effectively controls the growth of *Salmonella typhimurium* and inhibits the expression of the ATP synthase alpha chain protein during ATP production [115]. Furthermore, it inhibits the cell division of *Bacillus cereus* [116]. As EOs are inherently volatile and maintain a relatively short residence time, it is more common to use a slow-release form of CEO (e.g., microencapsulation technology) and then apply it to the preservation of edible mushrooms. In a study by Eliezer Louis et al., CEO microencapsulation was combined with paper-based materials to develop a bioactive paper for the preservation of edible mushrooms. It significantly inhibited the aging process of *A. bisporus* during cold storage, which was related to the antioxidant capacity of cinnamaldehyde and its antimicrobial capacity by reducing the droplet size to achieve uniform dispersion within the coating [84]. In addition, Shao et al. prepared bioactive packaging materials using starch/CEO microcapsules as a coating, which had good permeability and antibacterial properties, prevented water condensation and spoilage caused by water imbalance, and was successful in extending the shelf life of foods with high water content, such as *A. bisporus* [85]. Meanwhile, Zhang et al. prepared CEO-MSNP/potato starch films by mixing CEO-MSNPs in a potato starch matrix. The CEO-MSNPs in the films inhibited the pathogenicity of *Trichoderma* sp. F and *Trichoderma annulate* C by inducing the accumulation of intracellular reactive oxygen species and disrupting the integrity of the cell membrane [112]. SEM showed that CEO-MSNPs effectively disrupted the mycelial morphology of both molds and interfered with their normal growth. The expression levels of NOx genes in *Trichoderma* sp. F and *Trichoderma annulate* C significantly increased after treatment with CEO-MSNP (*p* < 0.01). One of the mechanisms of inhibition of these organisms by CEO-MSNPs may lie in the upregulation of nox1 and nox2 genes to produce large amounts of ROS [101,112]. Nair et al. prepared maize alcohol-soluble protein/ethyl cellulose hybrid nanofibers in different ratios while encapsulating CEO into electrospun fibers to preserve *A. bisporus*. This treatment effectively preserved the moisture and antioxidant capacity, enhanced antioxidant enzyme activity, reduced browning enzyme activity, delayed aging, and improved the shelf life of *A. bisporus* [117]. Pan et al. prepared a cross-linked electrospun polyvinyl alcohol/CEO/β-cyclodextrin (CPVA-CEO-β-CD) nanofiber film for the sustained release of antibacterial drugs by encapsulating CEO in PVA- and β-CD-based fibers using electrostatic spinning, which could inhibit Gram-positive and Gram-negative bacteria and effectively extend their shelf life [99].

Whether for the CEO microcapsules of Shao et al. [85] or the CEO-MSNPs of Zhang et al. [112], or the nanoemulsions containing different concentrations of cinnamaldehyde produced by Eliezer et al. [84], the membrane permeability, ROS content, cellular component leakage, cellular component leakage, soluble sugars, and malondialdehyde (MDA) content after the addition of CEO at the lowest inhibitory concentration level were significantly changed. By incorporating cinnamaldehyde nanoemulsions into the coatings, the quality of the mushrooms was significantly improved, reducing the respiration rate, weight loss, PPO activity, and *Pseudomonas* population, and increasing the retention of the hardness, color, total polyphenols, and antioxidant capacity of the mushrooms—positive effects that may be related to the antioxidant capacity of CIN and improving its antimicrobial capacity by achieving uniform dispersion within the medium, thus significantly extending the shelf life of the mushrooms.

#### 3.1.4. Cumin Essential Oil (CUEO)

Cumin (*Cuminum cyminum* L.) is an aromatic plant naturally cultivated in many countries, such as Iran, India, China, Japan, Morocco, and Egypt [118]. Cumin is the second most popular spice in the world after black pepper and has many applications in food [119]. CUEO, with its good antibacterial properties, is also used in the preservation of edible mushrooms [93,94,120]. Roghayeh Karimirad et al. prepared CUEO-chitosan nanoparticles (CUEO-CSNPs) using subcellular-sized chitosan trimeric phosphate (CS-TPP) as a controlled-release system to encapsulate EOs, a treatment that induced the synthesis of catalase (CAT), glutathione reductase (GR), and ascorbic acid and slowed down the increase in peroxidase activity [93]. The experimental data showed that the highest CAT and GR activities were observed in the samples containing CUEO-CSNPs after 15 days of storage. In contrast, the POD activity of the control samples peaked at the end of storage. Interestingly, the POD levels of the CUEO-CSNP-treated samples increased by 17.13% after 20 days compared to the initial levels of the enzymes mentioned above. At the end of storage, the levels of ascorbic acid in the CUEO-CSNP-treated samples were significantly higher than those in the control samples. Fumigation of *A. bisporus* with cumin seed oil extended its shelf life by 15 days with no significant reduction in antioxidant activity. Meanwhile, storage of *A. bisporus* at 4 °C using chitosan nanoparticles as a cumin oil delivery system reduced the microbial population, facilitated the maintenance of the antioxidant capacity, improved the tissue hardness, and inhibited the formation of brown patches compared to conventional packaging [93,94].

#### 3.1.5. Thyme Essential Oil (TEO)

As a food-grade preparation, TEO has broad-spectrum antibacterial activity against pathogenic bacteria [121]. Zhu et al. evaluated the effects on the postharvest quality and antioxidant enzyme activity of *Pholiota nameko* mushroom by developing SA-based composite coatings enriched with or without TEO, lactobacillin, and L-cysteine for the preservation of *P. nameko*. In all experimental groups, the addition of 1% (V/V) TEO, 0.3 g/L cysteine, and 0.4 g/L lactic acid streptococin effectively controlled the senescence, growth of aerobic mesophilic bacteria, and deterioration of quality of *P. nameko* and they found that the SA-based coating effectively reduced the weight loss, cap opening, browning, MDA content, and total soluble phenol content compared to the untreated control. In addition, the appearance of peak SOD, POD, PPO, and CAT activities was delayed after the coating treatment. During storage, ascorbic acid, soluble sugars, protein, and PAL activities were higher in treated mushrooms than in untreated ones [122]. Thus, this demonstrates the positive effect of TEO on the storage and preservation of edible mushrooms.

#### 3.1.6. Turmeric Essential Oil (TUEO)

Turmeric (*Curcuma longa*) is a medicinal plant of the Zingiberaceae family and is the most common spice used in cooking and many health products [123,124]. The biological activities of TUEO, including antitumor, antibacterial, anti-inflammatory, and antioxidant properties, have been demonstrated in several studies [125,126]. Valizadeh et al. evaluated the effect of TUEO and TUEO-incorporated chitosan nanoparticles (TUEO-CSNPs) on the shelf life of white mushrooms by inducing an increase in CAT and SOD activities. Mushrooms packed with TUEO-CSNPs and fumigated with TUEO were found to be significantly firmer and showed lower changes in color and microbial counts at day 15 of storage compared to untreated samples. In addition, TUEO-CSNP treatment showed the highest SOD, CAT, total phenolic, and ascorbic acid content and the lowest PPO activity (*p* < 0.01) [127]. Although the use of nanoparticles in Valizadeh’s method would impose an additional cost on the packaging, the extended shelf life, maintenance of mushroom quality, and reduced crop losses can justify this additional cost.

#### 3.1.7. *Satureja khuzistanica* Essential Oil (SKO)

*Satureja khuzistanica* is a traditional herb endemic among the nomadic population of Southwestern Iran (including the provinces of Ilam, Lorestan, and Khuzestan) [128,129]. It is used in herbal tea for its analgesic, antiseptic, and anti-inflammatory properties, especially for toothache problems [128]. It also has antispasmodic, antidiarrheal, vasodilatory, hypolipidemic, and antioxidant properties, as well as antifungal, antiviral, and antibacterial properties [130,131]. SKO is commonly used as a natural antioxidant and antimicrobial agent and as an antifungal agent in liquid culture media [132,133]. Nasiri et al. determined the effect of baicalin gum (TG) coatings containing different concentrations (100, 500, and 1000 ppm) of saturated SKO on the storage and preservation of *A. bisporus* mushrooms to maintain tissue hardness and sensory quality, reduce the number of microorganisms, and decrease the rate of decomposition of phenolic compounds and ascorbic acid to prolong their shelf life [91]. The results indicated that treatment with SKO containing TG (TGSEO) maintained 92.4% tissue hardness and reduced the number of microorganisms such as yeast, molds, and *Pseudomonas* compared to uncoated samples. In addition, the TGSKO-coated mushrooms showed a 57.1% reduction in the browning index (BI) and significantly higher total phenolic content (85.6%) and ascorbic acid accumulation (71.8%) than the control group, making it more efficient than the TG coating alone [91]. 

### 3.2. Antibacterial Mechanisms of EOs as Preservatives

**Figure 3:**
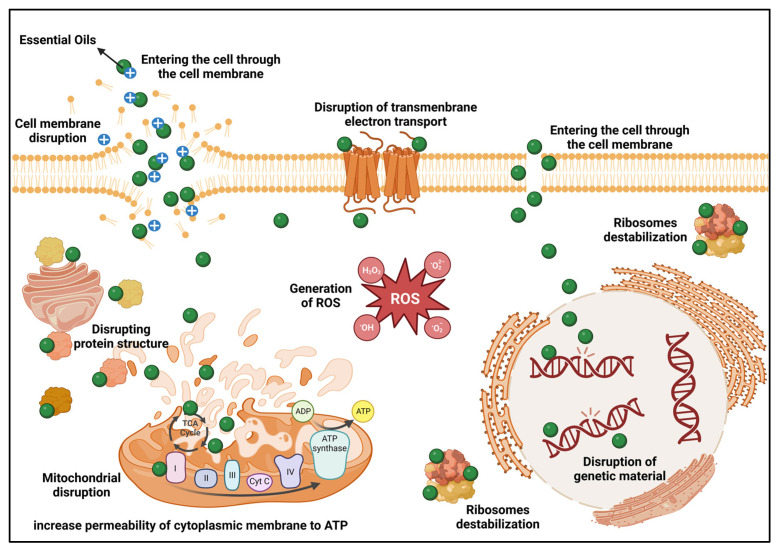
Bacteriostatic mechanisms of essential oils as preservatives for edible mushrooms. Created with BioRender.com.

The mechanism of action of EOs as preservatives for edible mushrooms is mainly due to their superior antibacterial mechanisms. Essential oils and EO slow-release systems release more ions and disperse them in the environment around the cell walls of pathogenic bacteria, where they effectively modify their pathogenicity. Direct interaction with the cell membrane produces metal ions and disrupts the cell membrane, disrupting the transmission signal to the cell membrane. At the same time, the electron transport chain is inhibited, which disrupts protein function within the bacterium and causes oxidative stress and DNA damage, resulting in the disruption of cell membrane formation [79,134] (Figure 3).

In summary, the use of EOs to preserve edible mushrooms is widespread and has good preservation effects. However, the current research is still focused on the effects on preservation, and there is a lack of studies on the mechanisms of EOs as a preservative for different applications in the edible mushroom industry. First, although there have been studies on the microbial inhibition of EOs, the in vitro strains selected for inhibition are still typically Gram-positive (*S. aureus*) and Gram-negative (*E. coli*), which may lack specificity. For example, it would be more meaningful to select *Listeria monocytogenes*, which has a relatively high rate of infestation of *Flammulina velutiper* and *Pleurotus eryngii*, for relevant mechanistic studies when conducting research on the inhibition effect and mechanism. Second, there is a lack of investigation of the mechanism of EOs from an energetic point of view. Whether the use of EOs changes the structure of mitochondria and modifies enzymes and related energetic substances in the respiratory chain still needs further investigation in terms of the respiratory effects of edible mushrooms.

### 3.3. Application of Plant Extracts in the Preservation of Edible Mushrooms

The main causes of edible mushroom decay and spoilage are oxidation reactions and bacterial growth [33,135]. The food industry has attempted to address these issues by incorporating additives into food products or implementing different packaging technologies. Packaging containing plant extracts is a popular direction for research to obtain biopolymeric food packaging. This is unique because it breaks down quickly into organic waste and can be easily infused with plant extracts, which results in active packaging with selective antibacterial and antioxidant food preservation effects that have positive implications for food storage and preservation [136]. According to numerous studies, plant extracts are promising alternatives to active food packaging due to their numerous properties, especially their ability to act as antioxidants and exert antibacterial activity [137,138] (Table 2).

Edible coatings are generally composed of polysaccharides, proteins, and lipids, which are generally derived from a variety of agricultural products and food processing wastes and by-products [38]. The polysaccharide coatings most commonly used are chitosan, pectin, carrageenan, gum, alginate, agar, and cellulose, and starch derivatives. Lecithin in lipids is mostly used as an emulsifier to dissolve oil-containing paints [144]. Edible coatings usually retard the shelf life of edible mushrooms by impeding gas exchange properties [145].

#### 3.3.1. Application and Mechanism of Action of Plant Polysaccharides in the Preservation of Edible Mushrooms

**Figure 4:**
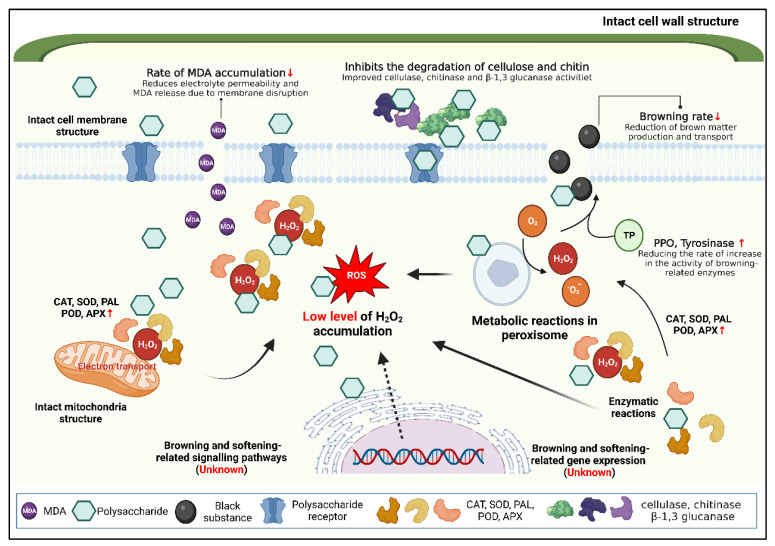
Mechanism of action of plant polysaccharides in the preservation of edible mushrooms. Created with BioRender.com.

*Oudemansiella radicata* polysaccharide (ORWP) is a natural polysaccharide extracted from the cysts of *Oudemannia*, a heteropolysaccharide with monosaccharides such as glucose, mannose, and galactose as the main components. Intriguingly, ORWP exhibits a wide range of actions, such as antifungal, antioxidant, and hepatoprotective activities [146,147]. Meanwhile, ORWP is a non-toxic and environmentally friendly compound tested as a coating agent for the preservation and shelf life extension of fresh flat mushrooms. Previous studies have found that it provides an effective barrier to the external environment, resulting in improved weight maintenance, delayed aging, and improved postharvest quality [146]. Liu et al. assessed the extent to which an ORWP coating improved the key quality characteristics of shiitake mushrooms during storage at 4 °C for 18 days and found that the use of the ORWP coating improved the retention of nutrients and flavor components, reduced MDA production, increased antioxidant enzyme activity, and improved the physical structure of shiitake mushrooms. Additionally, the ORWP coating was found to retard the softening of the mushroom due to the inhibition of cell wall hydrolases and, thus, the degradation of cellulose and chitin [142]. Shiitake mushroom polysaccharide (LEP) is a natural polysaccharide extracted from the stem of the shiitake mushroom and consists mainly of glucose [148], and it shows a wide range of actions, such as antioxidant and hypoglycemic effects [149]. It improved the weight retention, delayed the decay, and improved the postharvest quality of shiitake mushrooms by providing an effective external environmental barrier. Guo et al. investigated the effect of a polysaccharide coating on the browning and softening of shiitake mushrooms by showing that LEP increased the activities of POD, CAT, SOD, APX, and PAL and significantly reduced the accumulation of hydrogen peroxide compared to the control [148]. In addition, during storage, LEP treatment maintained the high antioxidant activity of the mushroom and inhibited the activity of browning-related enzymes (polyphenol oxidase and tyrosinase) to reduce browning. Furthermore, it maintained high levels of cellulase, chitinase, and β-1,3 glucanase to improve softening during storage [148]. The mechanism of action of plant polysaccharides in the preservation of edible mushrooms is illustrated in Figure 4.

#### 3.3.2. Application and Mechanism of Action of Melatonin in the Preservation of Edible Mushrooms

**Figure 5:**
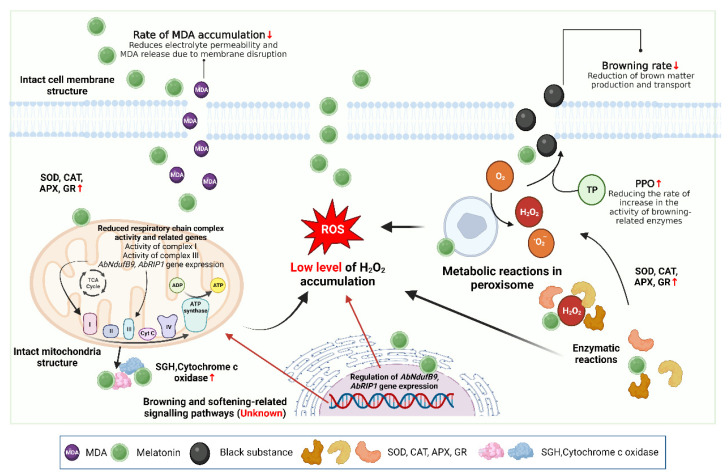
Application and mechanism of action of melatonin in the preservation of edible mushrooms. Created with BioRender.com.

Melatonin (N-acetyl-5-methoxytryptamine; MT) is a pleiotropic molecule commonly found in nature [150]. It plays a multifaceted role not only in humans and animals but also in the growth and development of plants [151]. In plants and bacteria, the best-known function of MT is associated with ameliorating abiotic stresses caused by various factors such as drought, radiation, extreme temperatures, and chemical stress, all of which have been reported to promote the production of ROS [152]. Many studies have shown that MT exhibits important defensive effects against various abiotic stresses in plants and animals [153,154]. Melatonin can mediate selenium-induced tolerance to Cd stress in tomatoes via Cd detoxification and can ameliorate water deficit stress in grapes through antioxidant metabolites [154,155]. Li et al. investigated the effect of MT in delaying aging by regulating electron leakage in postharvest *A. bisporus* and found that 0.1 mM MT treatment increased the antioxidant system and significantly inhibited electron leakage. Furthermore, MT had a clear and beneficial effect in maintaining higher ATP, cytochrome c oxidase (Cyt C), and energy levels, increasing the oxidative phosphorylation and efficiency of mitochondria, and delaying the senescence process in postharvest white mushrooms [49]. In contrast, Gao et al. focused on enhancing the tolerance of mushrooms to Cd through antioxidant-related metabolites and enzymes to verify the ameliorative effect of MT on Cd-induced oxidative stress. MT can promote the antioxidant activity of *Volvariella volvacea* via amino acid metabolism, glutathione metabolism, redox processes, detoxification, and cellular oxidant detoxification, suggesting that exogenous MT has a protective effect on Cd-induced oxidative stress in edible mushrooms [143]. Thus, MT contains several active compounds that have a selective effect on the autoxidation and antibacterial pathways in the preservation of edible mushrooms, making it a good choice for the storage and preservation of edible mushrooms [154]. The application and mechanism of action of MT in the preservation of edible mushrooms are shown in Figure 5.

#### 3.3.3. Application and Mechanism of Action of Exogenous Energy Substances in the Preservation of Edible Mushrooms

**Figure 6:**
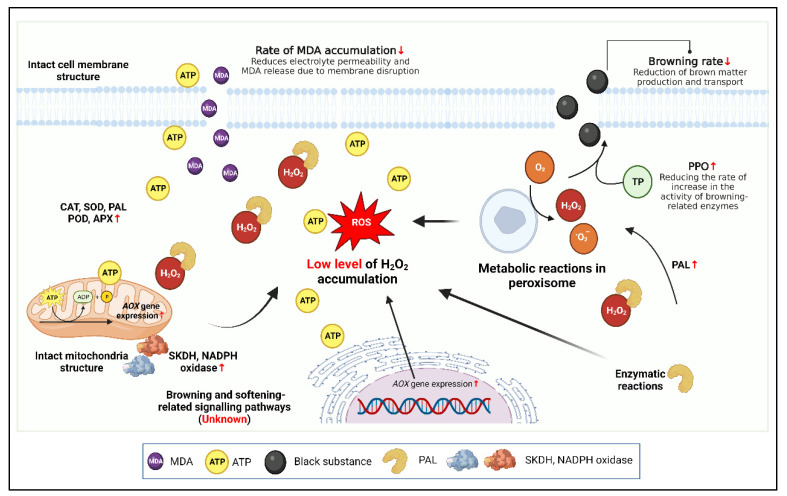
Application and mechanism of action of exogenous energy substances in the preservation of edible mushrooms. Created with BioRender.com.

Exogenous adenosine triphosphate (ATP) treatment has been used as an innovative method to improve stress, delay senescence, and preserve quality in horticultural crops during postharvest storage [156]. It may be an effective way to reduce electrolyte leakage and MDA accumulation, protect membrane integrity, and facilitate delayed aging. A study by Aghdam et al. demonstrated reduced browning and preservation of quality, increased phenol accumulation, and improved ability to scavenge ·DPPH in ATP-treated shiitake mushrooms. In addition to having higher endogenous phenol and MT accumulation, they showed a lower H_2_O_2_ accumulation capacity and could scavenge ROS [140]. The application and mechanism of action of exogenous energy substances in the preservation of edible mushrooms are presented in Figure 6.

#### 3.3.4. Application of Other Plant Extracts in the Preservation of Edible Mushrooms

Gamma-aminobutyric acid (GABA) is a neuroactive inhibitory neurotransmitter molecule with beneficial effects on human health [157,158]. Therefore, GABA has received considerable attention from the food and pharmaceutical industries as a safe and environmentally friendly functional bioactive molecule for health promotion, and, as a result, GABA-enriched foods have been commercialized worldwide [159]. GABA is an endogenous signaling molecule that plays an important role in plant growth, pH regulation, nitrogen storage, osmoregulation, ROS scavenging, and defense against abiotic and biotic stresses [160,161]. GABA biosynthesis occurs mainly in the GABA shunt pathway. Glutamic acid decarboxylase (GAD), GABA transaminase (GABA-T), and succinic semialdehyde dehydrogenase (SSADH) are involved in GABA metabolism [162]. It was demonstrated that exogenous GABA increased GAD gene expression, thereby increasing glutamate production from endogenous GABA, improving the antioxidant capacity of mushrooms, and reducing oxidative stress damage in edible mushrooms during storage. The results from the study of Shekari et al. revealed that GABA-treated mushrooms had lower rates of browning and weight loss and maintained their hardness better than untreated mushrooms [139]. GABA treatment maintained membrane integrity and reduced membrane lipid peroxidation. Treated mushrooms exhibited lower electrolyte leakage rates and MDA content. ASA content was higher in GABA-treated mushrooms, while ROS and H_2_O_2_ content were lower than in the untreated group. The higher accumulation of total phenolics in the treated mushrooms increased their antioxidant capacity. The treated mushroom strains showed higher PAL activity and gene expression and lower PPO activity [139].

In addition to the above investigations, agricultural waste extracts have also been studied for their application in preserving edible mushrooms. Pistachio shells (PGH) are a cheap and abundant natural source of useful compounds (>104.0 × 10^6^ kg) from agricultural waste, and pistachio shells make up 35–45% of the whole fruit [163]. The high phenolic and antioxidant capacity of PGH suggests that it may be a cost-effective source of bioactive compounds with health-protective potential [164,165,166,167]. Its main components are anaerobic acid (31.98 g kg^−1^), fatty acids (15.00 g kg^−1^), and phytosterols (19.20 g kg^−1^). The main phenolic compounds of pistachio green shell (*Pistacia vera* L., variety Bronte) are gallic acid, 4-hydroxybenzoic acid, protocatechuic acid, naringin, eriodictyol-7-O-glucoside, isorhamnetin 7-O-glucoside, quercetin-3-O-rutinoside, isorhamnetin 3-O-glucoside, and catechins [163]. Fattahifar et al., investigated the inhibitory activity of pistachio husk extract (PGHE) on mushroom tyrosinase and showed that PGHE-treated mushrooms had a lower BI than other samples. Pistachio green hull extract (PGHE) maintained the hardness (13.9% higher than the control) and whiteness of the mushrooms. It kept the organoleptic properties of the mushrooms within an acceptable range throughout storage and they had higher phenolic content (14.4%) and antioxidant activity (4.5%) than the control samples at the end of storage [141]. Pistachio shell extract is a novel natural tyrosinase inhibitor for reducing browning and related enzymatic browning reactions in edible mushrooms, while its cost advantage can distinguish it among the wide range of food preservation methods.

## 4. Perspectives and Conclusions

Research related to the preservation of mushrooms by EOs and plant extracts in recent years has shown that, although effective in preserving some of the key quality attributes of edible mushrooms, these preservation techniques have their drawbacks. For example, some essential oil treatments can significantly extend the shelf life of mushrooms; however, they lead to texture changes and discoloration, which affects customer acceptance. The use of nanoparticles for the controlled release of EOs in mushrooms maintains their quality during storage and extends their shelf life but adds additional costs in terms of packaging. This suggests that the area involving the use of EOs and plant extracts for preserving mushrooms still needs to be developed. In the future, it is important to further investigate the mechanisms of quality fission during storage and preservation, such as browning, softening, and lignification, through multi-omics techniques, and to study the potential molecular mechanisms of gene regulation during preservation, so that the problem of the postharvest quality deterioration of edible mushrooms can be addressed at the molecular level. In addition, a combination of new and traditional technologies can be used to improve the postharvest quality of edible mushrooms, combining various technologies at a lower capital cost or shorter processing time to further enhance the postharvest quality of mushrooms.

## Figures and Tables

**Table 1:** Essential oils (EOs) used for the control of postharvest preservation of mushroom.

Mushroom	Essential Oil Species	Progress	Storage Time	Preservation Effect	Ref.
*Agaricus bisporus*	Oil species: Eucalyptus leaf essential oil Source: Ji’an Guoguang Spice Factory	Group: (1) film without EOM (2) film with 5 mL/100 mL EOM (3) film with 10 mL/100 mL EOM (4) film with 15 mL/100 mL EOM (5) film with 20 mL/100 mL EOM	12 d	Optimal processing: Film with 15 mL/100 mL EOM Preservation effect: -Ensuring the integrity of the packaged food: Tensile strength (16.1 MPa); elongation at break (165%); -Inhibiting the bacteria: Size of film inhibition circle: Escherichia coli (14 mm); Staphylococcus aureus (23 mm); Trichoderma (47 mm)	[86]
*Agaricus bisporus*	Oil species: Cinnamon essential oil (CEO) Source: Shanghai Aladdin Biochemical Technology Co.	Storage temperature: 20 ± 3 °C Group: (1) packed with ZE-11 nanofibers (2) packed with ZE-11CEO nanofibers (3) without packing film (control group)	6 d	Optimal processing: packed with ZE-11CEO nanofibers Preservation effect: -Maintain the most uniform effect among composite nanofibers: the lowest porosity: 68.47% ± 4.3% -Maintain relatively good firmness: the degradation of the cell wall was slowed down and cell respiration was inhibited due to the antioxidant and bacteriostatic effects of CEO -The quality of mushrooms was regarded as good: the highest L* value on the 6th day: 75.31 ± 0.23; the lowest BI: 43.87 ± 0.36 -Maintain good sensorial property: showed a good appearance on the 6th day	[87]
*Agaricus bisporus*	Oil species: Cinnamaldehyde essential oil Source: Aladdin Inc. (Shanghai, China)	Storage temperature: 20 ± 2 °C Group: (1) packed with an LDPE film (2) packed with zein/SDS nanofibers (3) unpackaged	4 d	Optimal processing: packed with zein/SDS nanofibers Preservation effect: -Maintain good texture characteristics: firmness 195 g at 4 days -Maintain good appearance: L* (76.18 ± 0.20), a* (4.68 ± 0.18), b* (21.39 ± 0.05), ΔE (30.59 ± 0.08), BI (36.37 ± 0.06) at 4 days	[88]
*Agaricus bisporus*	Oil species: α-tocopherol with 98% purity and zein Source: Nanjing Dulai Reagent Co. Ltd.	Storage temperature: 4 °C Group: (1) no film packaging (control group) (2) CS film packaging (3) C/Z film packaging (4) C/Z/T film packaging	12 d	Optimal processing: C/Z/T film packaging Preservation effect: -Maintain the lowest respiration rate: the lowest O_2_ permeability and CO_2_ concentration -Delay dehydration: weight loss: 8.35% at the end of 12 d -Maintain higher firmness values -Inhibit the oxidation of phenolic compounds: the BI had 1.62-fold reduction versus control after 12 d of storage; CAT and SOD activity firstly increased and then decreased -Maintain the integrity: reduced MDA content to prevent lipid peroxidation -Maintain the integrity of the cell membrane: highest total phenolic content; the DPPH radical scavenging activity was significantly higher than control except for 6 d	[89]
*Agaricus bisporus*	Oil species: Lemon essential oil Source: Guangzhou Dakai Biotechnology Co. Ltd. (Guangzhou, China)	Storage temperature: 4 °C Storage relative humidity: 90% Group: (1) Control; (2) C/Z/L0 (LEO 0% (*w*/*w*)) of total solids; C/Z/L-3 (LEO 3%); C/Z/L-6 (LEO 6%); C/Z/L-9 (LEO 9%); C/Z/L-12 (LEO 12%) of total solids	12 d	Optimal processing: C/Z/L-6 film packaging: LEO at 6% (*w*/*w*) of total solids Preservation effect: -Inhibiting the bacteria: size of film inhibition circle: *Escherichia coli* (12.13 mm); *Staphylococcus aureus* (26.03 mm) -Delaying the aging and quality deterioration of mushrooms: hardness (573.34 g); springiness (0.73 dimensionless); cohesiveness (0.58 dimensionless); chewiness (215.73 g); total plate count (6.25 log10 CFU g^−1^)	[70]
*Agaricus bisporus*	Oil species: Oregano essential oil Source: obtained from Jiangxi Global Natural Flavor Co., Ltd. (Jiangxi, China)	Group: (1) sodium alginate (SA) films (2) 0.4 wt% OEO-MSNPs (3) 0.6 wt% OEO-MSNPs (4) 0.8 wt% OEO-MSNPs (5) 1.0 wt% OEO-MSNPs	4 d	Optimal processing: 0.8 wt% concentration of OEO-MSNPs Preservation effect: -Maintaining the film characteristics, ensuring the integrity of the packaged food: thickness (97.66 μm); water vapor permeability (8.50×10^−9^·g·d^−1^·m^−1^·Pa^−1^); opacity (5.20 dimensionless); tensile strength (9.39 MPa); Young’s modulus (75.82 MPa); elongation at break (27.22%); water contact angle (38.88 dimensionless)	[90]
*Agaricus bisporus*	Oil species: Cinnamaldehyde essential oil Source: Alg extracted from brown algae (A2033, Sigma-Aldrich, Chile) CIN purchased from Sigma-Aldrich Chile W252506)	Storage temperature: 4 °C Group: (1) uncoated mushrooms (2) mushrooms coated with Alg (3) Alg + 0.025 CIN (4) Alg + 0.05 % CIN (5) Alg + 0.1 % CIN	16 d	Optimal processing: Alg + 0.05 Preservation effect: -Maintaining the coating-forming emulsions’ characteristics: alginate (1 g/100 mL); glycerol (0.125 mL/g alginate); tween 80 (0.05 mL/100 mL); CIN (0.05 mL/100 mL) -Delaying the aging and quality deterioration of mushrooms: total polyphenols (17.2%); DPPH inhibition (46.8%); PPO activity (88.4%); weight loss (2.5%) at 16 days	[84]
*Agaricus bisporus*	Oil species: Cinnamon essential oil Source: Purchased from Saan Chemical Technology Co., Ltd.	Group: (1) Uncoated paper (2) Starch/microcapsules-coated paper (1:1) (3) Starch/microcapsule-coated paper (1:3) (4) Starch/microcapsule-coated paper (1:5) (5) CEO-coated paper	4 d	Optimal processing: Starch/microcapsule-coated paper (1:5) Preservation effect: -Maintaining the papers’ characteristics: basis weight (86.9 g/m^2^); thickness (0.105 mm); compactness (0.83 g/m^2^); permeability (8.50 μm/Paxs); Cobb_60_ (29.20 g/m^2^) -Delaying the aging and quality deterioration of mushrooms: APX activity (6.73 mol/min/g); GR activity (7.32 mol/min/g); weight loss (4.27%)	[85]
*Agaricus bisporus*	Oil species: Satureja khuzistanica essential oil (SEO) Tragacanth gum (TG) Source: SEO provided by a local commercial producer of plant essential oils, TG purchased from a local herbalist store	Storage temperature: 4 ± 1 °C Storage relative humidity: 90% Group: (1) control: water (2) TG: Tragacanth gum coating (3) TGSEO1: TG coating containing 100 ppm SEO (4) TGSEO5: TG containing 500 ppm (5) TGSEO10: TG containing 1000 ppm	16 d	Optimal processing: TGSEO5 Preservation effect: -Delayed the aging and quality deterioration of mushrooms, reduced respiration rate: weight loss: 2.2%; browning index (BI): 18.2; respiration rate: 74.15 mg CO_2_ kg^−1^ h^−1^ -Inhibit the softening of mushrooms: firmness: 8.3% -Maintain high antioxidant activity: total phenolic content (TPC): 85.6%; ascorbic acid content: 69.6%	[91]
*Agaricus bisporus*	Oil species: *C. aurantium* peel oil Source: Extracted from the dried peel of *C. aurantium*	Storage temperature: 4 °C Storage relative humidity: 95% Group: (1) Control (2) C. aurantium essential oil (CAEO) (3) C. aurantium essential oil-loaded chitosan nanoparticles (CAEO-CSNPs)	20 d	Optimal processing: CAEO-CSNPs Preservation effect: -Delayed the aging and quality deterioration of mushrooms: weight loss: 2%; L*value: 84.48 -Inhibit the softening of mushrooms: firmness: 13.94 N -Maintain high antioxidant activity: total phenolic content (TPC): 84.13 mg 100 g^−1^ FW; ascorbic acid content: decreased by 9.53% at 20 days compared to the first day -Inhibit enzyme activity: PPO: 24.38 U g^−1^ FW -Improve antioxidant capacity: based on DPPH assay, the antioxidant capacity reached 59.43% at 15 days	[92]
*Agaricus bisporus*	Oil species: Cumin seed oil (CEO) Source: Extracted from cumin (Cuminum cyminum) seeds	Storage temperature: 4 °C Group: (1) Control (2) CEO (3) CEO-CSNPs	20 d	Optimal processing: CEO-CSNPs Preservation effect: -Inhibit the activity of the enzyme: CAT and GR increased 2.35 and 3.47 times at 15 days, respectively, compared to their initial levels -Maintain high antioxidant activity: ascorbic acid (AA) content was decreased by 5.83% at 20 days compared to the initial content	[93]
*Agaricus bisporus* (cv. Sylvan A15)	Oil species: Cuminum cyminum oil (CEO) Source: Cumin seeds	Storage temperature: 4 °C Group: (1) untreated (2) CEO (3) CEO-CSNPs	20 d	Optimal processing: CEO-CSNPs Preservation effect: -Inhibit microbial growth: mesophilic bacteria (9.01 ± 0.098) (log10 cfu/g), psychrophilic bacteria (9.26 ± 0.10) (log10 cfu/g), yeasts and molds (9.26 ± 0.100) (log10 cfu/g) at 20 days -Maintain good texture characteristics: firmness 14 N at 20 days -Maintain good appearance: L* (84.72 ± 0.423), ΔE (18.29 ± 0.249), BI (22.56 ± 0.142) at 20 days; overall acceptability is high -SOD, APX activity is high, PPO activity is low: SOD activity 3.9 (U/g FW), APX activity 12 (U/g FW), PPO activity 15 (U/g FW) at 20 days -Strong antioxidant capacity: total phenolic content 0.65 (g kg^−1^ FW), DPPH radical scavenging capacity 65%, ABTS^+^ radical scavenging capacity 67%	[94]
cv. Sylvan A15	Oil species: Citrus aurantium essential oil (CAEO) Source: *C. aurantium* peel	Storage temperature: 4 °C Storage relative humidity: 90% Group: (1) chitosan:CAEO = 1:0.25 (2) chitosan:CAEO = 1:0.5 (3) chitosan:CAEO = 1:0.75 (4) chitosan:CAEO = 1:1 (5) chitosan:CAEO = 1:1.25	20 d	Optimal processing: ratio of chitosan to CAEO: 1:0.5 Preservation effect: -Maintain smaller hydrodynamic diameter in the aqueous medium: average size: 20–60 nm -Maintain maximum EE and LC values -Inhibit the growth of the studied microorganisms -Maintain higher antioxidant enzyme activity	[95]
*Tremella fuciformis*	Oil species: Roasted peanut skin extract Source: Local market (Daejeon, Korea)	Storage temperature: 20 °C Storage relative humidity: 60% Group: (1) the mixture (2) 0.25 g/100 mL PSE were added to the mixture (3) 0.5 g/100 mL PSE were added to the mixture (4) 1.0 g/100 mL PSE were added to the mixture	50 d	Optimal processing: 1.0 g/100 mL PSE were added to the mixture Preservation effect: -The highest TS: 23.99 Mpa, WVP: 4.99 × 10^−9^ g/m s Pa -Increase the surface hydrophobicity -TPC: 83.66 ± 0.57 mg GAE/g, the highest ABTS (92.85%) and DPPH (84.92%) -The incorporation of PSE did not affect the biodegradation rate of TFP films.	[96]
White mushroom	Oil species: Essential oil mix selected was eugenol/bergamot EO/grapefruit EO (60:20:20, volume, *v:v:v*) Source: Supplied by Lluch Essence S.L. (Barcelona, Spain)	Storage temperature: 4 °C Group: (1) CTRL (2) 100 μL L^−1^ essential oil (3) 125 μL L^−1^ essential oil	14 d	Optimal processing: 100 μL L^−1^ essential oil Preservation effect: -Delaying the aging and quality deterioration of mushrooms: pH (7.51); L* (Cap) (79.5); L* (Stipe) (75.5)	[97]
White mushroom	Oil species: Microencapsulated CAR (βCD-CARM)/sodium alginate (SA) films	Storage temperature: 23 ± 2 °C Storage relative humidity: 55 ± 5% Group: The mass ratios of the core-to-wall material are 1:4, 1:6, 1:8, 1:10, 1:12 Added to βCD-CARM 5 g/L, 15 g/L, 25 g/L, βCD (5 g/L), or CAR (5 g/L) as controls βCD-CARM/SA 15 g/L, 30 g/L, 60 g/L	12 d	Optimal processing: the mass ratios of the core-to-wall material: 1:10, βCD-CARM 15 g/L, βCD-CARM/SA 30 g/L Preservation effect: -Inhibited Trichoderma sp. effectively: highest EE of microcapsules: 89.65%; 15 g/L concentration of βCD-CARM showed effective antifungal activity -Better antifungal activity against Trichoderma sp.: βCD-CARM/SA film 30 g/L inhibition zone diameter: 9.83 nm -Highest thermostability: lowest decomposition rate -Strengthened ductility of the films -Increased SOD, CAT, and APX activities to alleviate oxidative damage	[98]
Mushroom	Oil species: Cinnamon essential oil (CEO, containing 80% trans-cinnamaldehyde) Source: Saan Chemical Technology Co., Ltd.	Storage temperature: 10 ± 0.5 °C Group: (1) Not packaged (2) PVA (3) CPVA-CEO (4) CPVA-0.5CEO-β-CD (5) CPVA-1.0CEO-β-CD (6) CPVA-1.5CEO-β-CD (7) Plastic wrap	5 d	Optimal processing: CPVA-1.5CEO-β-CD electrospun nanofibrous film Preservation effect: -Conductivity decreases and viscosity increases: conductivity: 272 mS/cm, viscosity: 455 m Pas -The hydrophilicity of PVA can be lowered: water contact angle value below 90° -Encapsulation of CEO inside the fiber structure permitted the compound to be effectively released -Improved the antimicrobial effect: lowest total colonies; mushrooms were intact and edible	[99]
	Oil species: Cajuput essential oil (CjEO) Source: Hi-media Laboratory (Mumbai, India)	Storage temperature: 4 ± 1 °C Group: (Percent nanoencapsulation efficiency (NE) and loading efficiency (LE) of CjEO-CSNP prepared at different ratios of CS:CjEO) 1:0.0; 1:0.2; 1:0.4; 1:0.6; 1:0.8; 1:1.0	5 d	Optimal processing: 1:1.0 (*w*/*v*) of CS to EO Preservation effect: -Increasing trend of encapsulation efficiency and loading capacity: the NE and LE values ranged from 45.86 to 92.26% and 0.69 to 8.87% -Reducing oxidative degradation of mushrooms: extract coated with CjEO-CSNP showed significantly (*p* < 0.05) higher activity (54.06, 53.35, and 50.39%)	[100]
*Agaricus bisporus*	Oil species: Cinnamon essential oil (CEO) Source: Purchased from Guoguang Co., Ltd. (Jilin, China)	Group: (1) The potato starch films (S0) (2) MSNPs/potato starch films (S1) (3) MSNPs-CEO/potato starch films (S2)	7 d	Optimal processing: MSNPs-CEO/potato starch films (S2) Preservation effect: -Reduced the crystallinity degree of films: S2: 47% (S0: 63%) -Improved the mechanical properties of the films: water vapor transmission rate (644.41 g·d^−1^·m^−2^); oxygen transmission rate (4.86 g·d^−1^·m^−2^); tensile strength (56.12M·Pa); elongation at break (50.00%); thickness (25.93 μm)	[101]
*Volvariella volvacea*	Oil species: CO, PO Source: CO was purchased from Macleans Biochemical Technology Co., Ltd.; PO was purchased from Luyuan Natural Spice Oil Refinery	Storage time: 16 ± 1 °C Group: (1) No film packaging (control) (2) PLA/PBAT/TPS (3) PLA/PBAT/TPS-PO (4) PLA/PBAT/TPS-CO	4 d	Optimal processing: PLA/PBAT/TPS-PO Preservation effect: -Stronger antioxidant capacity: low PPO activity; the highest TPCs; higher hardness; lower TSS value; free radical DPPH (41%) -Maintaining cell integrity: the smallest T23 (100–1000 ms) peak area -Inhibiting the activity of the enzyme: lower autolysis rates, increased to 42.3% eventually; the RWL showed the strongest inhibitory effect (*p* < 0.05) -Inhibiting the bacteria: a significant decrease (*p* < 0.05) in the bacterial counts	[102]
*Agaricus bisporus*	Oil species: Cinnamon essential oil (CEO) Source: Guoguang Co., Ltd. (Jilin, China)	Storage temperature: 28 °C Group: (1) Potato starch films (2) MSNP/potato starch films (3) MSNP-CEO/potato starch films	48 h	Optimal processing: MSNP-CEO/ potato starch films Preservation effect: -Strong antibacterial property: bacteriostatic circle of species is the largest	[101]

**Table 2:** Extracts used for the control of postharvest preservation of mushroom.

Mushroom	Extraction	Progress	Storage Time	Preservation Effect	Ref.
*Agaricus bisporus*	Name: Exogenous γ-aminobutyric acid (GABA)	Treatment temperature: 20 °C Treatment time: 5 min Storage time: 4 ± 0.5 °C Group: (1) GABA 0 mM (control) (2) GABA 0.01 mM (3) GABA 0.1 mM (4) GABA 1 mM (5) GABA 10 mM	15 d	Optimal processing: 0.1 mM GABA Preservation effect: -Maintain high quality features: Cap browning 25%, weight loss 2.7% at 15 days. -Maintain texture characteristics: Firmness 11 N at 15 days. -Maintain low membrane lipid peroxidation: Electrolyte leakage 18.4%, MDA content 9.6 μmol kg^−1^ FW at 15 days. -Strong antioxidant capacity: Phenolic compound content 1.5 mg GAE kg^−1^ FW, AsA content 24.3 mg kg^−1^ FW, GABA accumulation 250 μmol kg^−1^ FW, GAD gene expression 1.6 at 15 days. -PAL activity is high and PPO activity is low: PAL activity 26.0 U kg^−1^ protein, PPO activity 52.8 U kg^−1^ protein, at 15 days.	[139]
*Agaricus bisporus*	Name: Exogenous adenosine triphosphate (ATP) H_2_O: 50 mg per mL	Treatment temperature: 25 °C Treatment time: 5 min Storage time: 4 °C Relative humidity: 80–90% Group: (1) Distilled water (control) (2) 250 µM ATP (3) 500 µM ATP (4) 750 µM ATP (5) 1000 µM ATP	18 d	Optimal processing: 750 µM ATP Preservation effect: -Maintain high quality features: Cap browning 35 at 18 days. -MDA accumulation is low: MDA accumulation 22.5 mmol kg^−1^ at 18 days. -SKDH enzyme, PAL activity is high, NADPH oxidase enzyme, PPO activity is low: SKDH enzyme activity 14 mkat kg^−1^ protein, PAL activity 15 mkat kg^−1^ protein, NADPH oxidase enzyme activity 22 μmol O_2_^−^ kg^−1^ protein min^−1^, PPO activity 4.5 mkat kg^−1^ protein at 18 days.	[140]
*Agaricus bisporus*	Name: Pistachio green hull extract	Treatment temperature: 22 °C Pressure: 0.025 MPa Treatment time: 900 s Storage time: 4 °C Group: (1) PGHE: pistachio green hull extract (0.05 *w*/*v*%) (2) MET: sodium metabisulfite (0.2 *w*/*v*%) (3) ASC-CIT: mixture of ascorbic acid (0.1 *w*/*v*%) and citric acid (0.5 *w*/*v*%) (4) Control: distilled water	10 d	Optimal processing: PGHE Preservation effect: -Maintain high quality features: Protein content (25.5%), dry matter (7.2%), texture (10.7 N), L* (83.6), browning index (17.4) at 10 days. -Antioxidant capacity (AOC) is high: Total phenolic content (5.3 g GAE kg^−1^), DPPH radical scavenging activity (88.1%) at 10 days. -Inhibit microbial growth: Mesophilic microorganism count 5.73 log CFU g^−1^ at 10 days.	[141]
*Agaricus bisporus*	Name: A600605-0005 melatonin	Storage temperature: 3 ± 1 °C Storage relative humidity: 90–95% Group: (1) Control (2)–(4) Dipped in 0.05, 0.1, and 0.2 mM melatonin	12d	Optimal processing: 0.1 mM melatonin Preservation effect: -Maintain good quality and damp electron leakage: electron leakage: increased by 1.4-fold. -Delay aging: endogenous melatonin content: 26.37 ng g^−1^; ATP level: reduced 28.4%; energy charge: reduced 37.5%.	[49]
*Lentinula edodes*	Name: Oudemansiella radicata water-soluble polysaccharides (ORWP)	Storage temperature: 4 °C Storage relative humidity: 85–90% Group: (1) Control: washed with water, and given no coating (2)–(5) ORWP5: 5, 10, 15, and 20 g L^−1^ ORWP coating	18 d	Optimal processing: ORWP10 Preservation effect: -Delayed the aging and quality deterioration of mushrooms: Weight loss: 3.50%; softening: 34.48%; ΔE: 8.03. -Alleviated oxidative injury: MDA content: 1.47 μmol kg^−1^. -Maintain high antioxidant activity: Soluble protein: 1.82 g kg ^−1^; carbohydrate content: 13.96 g kg ^−1^; ascorbic acid: 0.022 g kg ^−1^; total phenol content: 1.99 g GAE kg^−1^; cellulose content: 8.36 g kg ^−1^; lignin content 32.90 g kg ^−1^; free amino acids: 27.03 g kg^−1^ dry weight; EUC value: 360.37; 5′- nucleotides: 4.38 g kg^−1^ dry weight. -Inhibit enzyme activity: Protease activity: 2.35U mg^−1^; SOD activity: 8.39 U mg^−1^; CAT activity: 13.76 U mg^−1^; PPO activity: 1.47 U mg^−1^ protein; POD activity: 4.21 U mg^−1^ protein; cellulase activity: 12.57 U mg^−1^ protein; chitinase activity: 10.02 U mg^−1^ protein; PAL activity: 0.68 U mg^−1^ protein.	[142]
*Lentinus edodes*	Name: Melatonin (MT)	Group: (1) Control (2) Cd-treated group: Cd2 (2 μM CdCl_2_), Cd5 (5 μM CdCl_2_), Cd8 (8 μM CdCl_2_) (3) Melatonin (MT)-treated group: MT50 (50 μM MT solution), MT100 (100 μM MT solution), and MT200 (200 μM MT solution) (4) Cd + MT-treated group (100 μM MT + 5 μM CdCl_2_)		Optimal processing: Cd + MT-treated group Preservation effect: -Improve the activity of antioxidant enzymes: CAT activity 20,500 U/mg prot, SOD activity 1.75 U/mg prot, POD activity 2.8 U/mg prot, GR activity 0.014 U/mg prot, APX activity 0.18 U/mg prot at 5 days. -Maintain high nutritional characteristics: Proline concentration 170 μg/g FW, total sugar concentration 9 mg/g FW at 5 days. -The level of endogenous ROS was significantly reduced: H_2_O_2_ content 175 pg/g FW, O_2_^–^ content 2.1 pg/g FW at 5 days.	[143]

## Data Availability

No new data were created or analyzed in this study. Data sharing is not applicable to this article.
